# Inborn Errors of Nucleoside Transporter (NT)-Encoding Genes (*SLC28* and *SLC29*)

**DOI:** 10.3390/ijms23158770

**Published:** 2022-08-07

**Authors:** Marçal Pastor-Anglada, Aida Mata-Ventosa, Sandra Pérez-Torras

**Affiliations:** 1 Departament de Bioquímica i Biomedicina Molecular, Institut de Biomedicina de la Universitat de Barcelona (IBUB), Universitat de Barcelona, 08028 Barcelona, Spain; 2 Institut de Recerca Sant Joan de Déu (IR SJD), Esplugues de Llobregat, 08950 Barcelona, Spain; 3 Centro de Investigación Biomédica en Red de Enfermedades Hepáticas y Digestivas (CIBER EHD), Instituto de Salud Carlos III, 28029 Madrid, Spain

**Keywords:** nucleosides, transporters, rare diseases, genetics

## Abstract

The proper regulation of nucleotide pools is essential for all types of cellular functions and depends on de novo nucleotide biosynthesis, salvage, and degradation pathways. Despite the apparent essentiality of these processes, a significant number of rare diseases associated with mutations in genes encoding various enzymes of these pathways have been already identified, and others are likely yet to come. However, knowledge on genetic alterations impacting on nucleoside and nucleobase transporters is still limited. At this moment three gene-encoding nucleoside and nucleobase transporter proteins have been reported to be mutated in humans, *SLC29A1*, *SLC29A3*, and *SLC28A1*, impacting on the expression and function of ENT1, ENT3, and CNT1, respectively. ENT1 alterations determine Augustine-null blood type and cause ectopic calcification during aging. ENT3 deficiency translates into various clinical manifestations and syndromes, altogether listed in the OMIM catalog as histiocytosis-lymphoadenopathy plus syndrome (OMIM#602782). CNT1 deficiency causes uridine-cytidineuria (URCTU) (OMIM#618477), a unique type of pyrimidineuria with an as yet not well-known clinical impact. Increasing knowledge on the physiological, molecular and structural features of these transporter proteins is helping us to better understand the biological basis behind the biochemical and clinical manifestations caused by these deficiencies. Moreover, they also support the view that some metabolic compensation might occur in these disturbances, because they do not seem to significantly impact nucleotide homeostasis, but rather other biological events associated with particular subtypes of transporter proteins.

## 1. Introductory Remarks on Inborn Errors Impacting on Nucleotide Metabolism

Cells rely upon nucleotide supply to maintain a plethora of biological functions. Purine and pyrimidine nucleotides can either be synthesized *de novo* by biochemically independent pathways, both fueled by glucose and amino acids, or be recycled from nucleosides and nucleobases using a complex network of salvage enzymes. As was recently reviewed, more than 30 defects impacting on purine and pyrimidine metabolism have been reported so far and nearly 20 are known to cause human disease [1,2].

**Figure 1:**
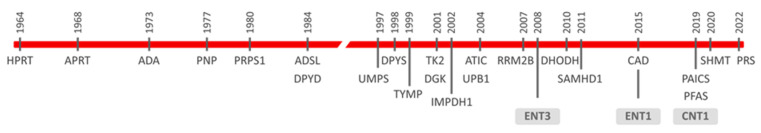
Chronogram of the discovery of rare diseases caused by mutations in genes encoding nucleotide metabolism-related proteins. Nucleoside transporters (ENT3, ENT1, and CNT1) are boxed. Full names of all enzyme acronyms are introduced in the text.

The first reported rare diseases were mostly associated with enzymes implicated either in nucleoside salvage or degradation [3]. Among them are Lesch–Nyhan disease [4], later shown to be caused by deficient hypoxanthine-guanine phosphoribosyltransferase (HPRT) activity [5], adenosine deaminase (ADA) deficiency [6] and purine nucleoside phosphorylase (PNP) deficiency [7]. Other genes of the nucleoside salvage/degradation network have been reported to be mutated since then (Figure 1). Moreover, quiescent cells appear to rely mostly on salvage pathways, and some of these dysfunctions (i.e., thymidine kinase 2 (TK2), deoxyguanosine kinase (DGK), thymidine phosphorylase (TYMP) deficiencies) determine mitochondrial DNA maintenance disorders, generally identified as mitochondrial DNA depletion and multiple deletions syndromes (MDDS) [8,9]. Considering that proliferating cells appear to be more dependent upon nucleotide *de novo* biosynthesis than salvage processes [10], intuitively, we might think that mutations impacting on the de novo machinery would translate into lethal phenotypes. However, it has been shown that this is not the case (Figure 1). Miller syndrome was clinically reported in the late 1970s but molecularly deciphered in 2010 [11]. Exome sequencing allowed the identification of mutations within the *DHODH* gene as being responsible for this syndrome. Dihydroorotate dehydrogenase (DHODH) is a key enzyme of the de novo pyrimidine biosynthetic pathway [12]. Carbamoyl-phosphate synthetase 2, aspartate transcarbamylase, and dihydroorotase (CAD) are highly regulated enzymes catalyzing the first three steps in pyrimidine nucleotide biosynthesis [13]. CAD deficiency was firstly characterized molecularly in 2015 [14], and since then up to 20 pathogenic mutations in the CAD gene have been reported [13]. The de novo purine nucleotide biosynthetic pathway is also mutated in humans [2]. Adenylosuccinate lyase (ADSL) deficiency was the first to be reported at the biochemical level in the 1980s. Indeed, its deficiency was deduced from the observation that three undiagnosed autistic patients had increased levels of the dephosphorylated forms of ADSL substrates in body fluids, and ADSL deficiency was tested at the enzymatic level [15]. A pathogenic point mutation in the ADSL gene was later reported in two of these patients [16]. More than 100 patients and around 50 pathogenic variants have been reported worldwide so far [2,17]. ADSL catalyzes the eighth step in the canonical synthesis of purine de novo synthesis and is also implicated in the conversion of the final product of this pathway, IMP, into AMP. Other genes followed, and in 2004 the first case of 5-aminoimidazole-4-carboxamide ribonucleotide formyltransferase/IMP cyclohydrolase (ATIC) deficiency in an infant showing AICA-ribosiduria and severe neurological alterations was reported [18]. ATIC is a bifunctional enzyme catalyzing the last two steps of the pathway which lead to IMP synthesis. Phosphoribosylaminoimidazole carboxylase and phosphoribosylaminoimidazole succinocarboxamide synthetase (PAICS) missense variants have also been identified recently [19]. PAICS is a bifunctional enzyme too, catalyzing the sixth and seventh canonical steps in the pathway. ATIC and PAICS deficiencies can be considered at this moment as extremely rare (five patients from four families and two from one single family have been reported, respectively) [2,19,20], although they are probably underdiagnosed. In this regard, the development of accurate, highly sensitive analytic methods, suitable for the quantification of metabolic intermediates in these pathways, is paving the way to identify novel inborn errors, as recently suggested for phosphoribosylformylglycinamidine synthase (PFAS), the enzyme catalyzing the fourth step in the purine de novo biosynthetic pathway [21].

A key metabolite in nucleotide metabolism is phosphoribosylpyrophosphate (PRPP). PRPP provides the ribose ring for purine and pyrimidine de novo nucleotide biosynthesis, but is also a co-substrate for key enzymes implicated in pyrimidine (uridine monophosphate synthetase, UMPS) and purine (hypoxanthine-guanine phosphoribosyltransferase, HPRT1, and adenine phosphoribosyltransferase, APRT) salvage [2]. As mentioned above, HPRT deficiency causes Lesch–Nyhan disease [5]. PRPP synthetase (PRS) catalyzes PRPP synthesis from ribose-5-P and ATP. PRS is feedback-inhibited by nucleotides and cell cycle-regulated via its phosphorylation by cyclin-dependent kinase 1 (CDK1) [22]. Therefore, fine tuning of PRS appears to be essential for the proper regulation of nucleotide homeostasis. Indeed, although rare, both gain- and loss-of-function variants in the *PRPS1* gene determine severe clinical phenotypes [2].

Thus, even though pathogenic variants in the enzyme machinery implicated in nucleotide metabolism, particularly those impacting on de novo biosynthesis, seem to be incompatible with life, it is clear that they occur, being rare but often extremely deleterious. Nevertheless, enzymes are not the only players in nucleotide homeostasis. A set of transporter proteins translocate nucleosides and nucleobases across biological membranes. Therefore, they are also candidates to regulate nucleotide availability.

The possibility that functional alterations in nucleoside transporter (NT) proteins could translate into human disease has not been studied in depth. With this contribution, we aim at providing an overview of the current, and still limited, knowledge on rare diseases associated with mutations in genes encoding nucleoside/nucleobase transporters. The pathogenic relevance of these mutations will be discussed based upon the current knowledge on the biology of these transporter proteins.

## 2. Nucleoside and Nucleobase Transporter Proteins

Two gene families encode NT proteins, *SLC28* and *SLC29*. The biochemical, regulatory and pharmacological properties of these proteins have been comprehensively reviewed previously [23,24,25,26]. Nevertheless, for a better follow-up of the contents of this review, we include a brief description of these transporter proteins below.

*SLC28* genes encode concentrative nucleoside transporter (CNT) proteins, CNT1, CNT2, and CNT3 (*SLC28A1*, *SLC28A2*, and *SLC28A3* genes, respectively), whereas the *SLC29* gene family encodes equilibrative nucleoside transporter (ENT) proteins, ENT1, ENT2, ENT3 and ENT4 (*SLC29A1*, *SLC29A2*, *SLC29A3*, and *SLC29A4* genes, respectively). CNT but not ENT orthologs are found in prokaryotes.

CNTs are high-affinity (apparent Km values in the low micromolar range), Na^+^-coupled concentrative nucleoside transporters showing some substrate selectivity. Indeed, CNT1 is a pyrimidine transporter, CNT2 a purine translocator which also accepts uridine as a substrate, and CNT3 shows broad selectivity for purine and pyrimidine nucleosides.

ENT1 and ENT2 are broad-selectivity, plasma membrane-facilitative nucleoside transporters. They share some substrate selectivity/specificity and apparent Km values for nucleosides higher than CNTs. ENT1 and ENT2 (but not CNT1, CNT2 or CNT3) have also been suggested to transport nucleobases, with ENT1 showing lower kinetic efficiency for these substrates [27]. The apparent affinities of ENT1 and ENT2 for nucleobases are lower than for nucleosides, although the transport efficiencies (*V*_max_/*K*_m_ ratios) of nucleoside and nucleobase transport appear to be similar. ENT1 is also expressed in mitochondria [28], where it can supply purine and pyrimidine deoxynucleosides to the matrix kinases TK2 and dGK, two enzymes whose deficiency, as mentioned above, result in MDDS [8,9]. ENT2 has also been found in intracellular membranes and by forming oligomers with truncated ENT2 variants generated by mRNA splicing localized in the nucleus [29]. Recruitment of ENT2 to the nucleus appears to be regulated and determined by the upregulation of the spliced variants under conditions of cell proliferation [29].

ENT3 is phylogenetically distant from the previous ENTs and relatively insensitive to ENT1/2 pharmacological inhibitors such as 6-S-[(4-Nitrophenyl)methyl]-6-thioinosine (NBMPR), dipyridamole and dilazep. In general trends, ENT3 nucleoside selectivity is relatively similar to ENT1 and ENT2, while also being able to translocate adenine. A major difference with the former ENTs is the fact that ENT3 utilizes a pH-sensitive mechanism for transport [30,31]. This makes sense considering that ENT3 is an intracellular transporter, shown to be expressed in lysosomes and mitochondria [30,32].

ENT4, also known as plasma membrane monoamine transporter (PMAT) shows the lowest homology with the previous ENT subtypes. Indeed, ENT4 may be H^+^-coupled and is basically a polyspecific organic cation transporter. Although it can translocate adenosine at an acidic pH, ENT4 does not seem to play any role in nucleotide metabolism and homeostasis [33,34].

More recently, the protein product of the *SLC43A1* gene has been identified as a purine nucleobase transporter called equilibrative nucleobase transporter 1 (ENBT1) [35]. ENBT1 can translocate adenine and guanine and, at least for adenine, its function is tightly coupled to the adenine salvage enzyme APRT [36].

In terms of tissue distribution, ENT1 and ENT3 show broad but variable expression both at the mRNA and protein levels, and could be considered almost ubiquitous. This is probably also the case for ENT2 [26], at least at the mRNA level, although protein expression appears to be less abundant than the other two subtypes. CNTs show a more restricted tissue distribution than ENTs when analyzed both at the mRNA and protein levels. They are mostly, but not exclusively, expressed in epithelial tissues, and in absorptive/reabsorptive tissues (i.e., gastrointestinal tract, nephrons), their expression is polarized, being located at the apical domain, thereby facilitating nucleoside vectorial flux across the epithelial barrier [26,37,38].

## 3. Nucleoside and Nucleobase Transporter Protein Variants and Disease

**Figure 2:**
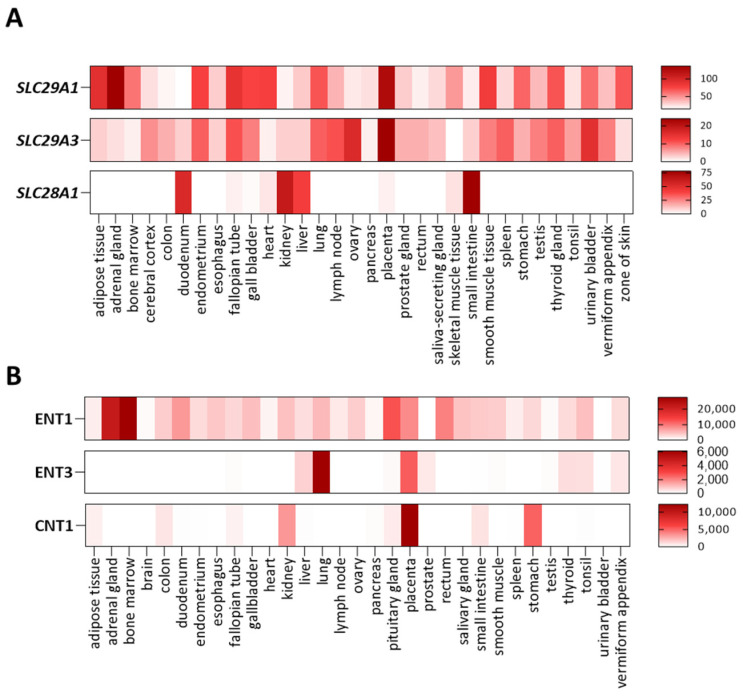
Nucleoside transporter expression patterns and substrate selectivity. Heat-maps showing the tissue expression of *SLC29A1* (ENT1), *SLC29A3* (ENT3) and *SLC28A1* (CNT1) at the mRNA (**A**) and protein (**B**) levels; data obtained from the EMBL-EBI database.

Nucleoside and nucleobase transporter genes are polymorphic to different extents, but at the moment of writing this review, genetic variants impacting on transporter function, leading to biochemical, cellular and clinical manifestations, have been exclusively identified for *SLC29A1* (ENT1), *SLC29A3* (ENT3), and *SLC28A1* (CNT1). Tissue expression patterns and substrate selectivity for these three membrane transporters are shown in Figure 2.

### 3.1. Genetic Alterations of ENT1. Augustine-Null Blood Type

Human ENT1 cDNA was obtained from placenta by means of expression cloning [39], and its structure was recently solved based upon human ENT1 crystals in a complex with two of its pharmacological inhibitors, dilazep and NBMPR [40]. ENT1 is a major modulator of adenosine levels inside and outside cells, thereby playing a role in purinergic signaling [41]. Based upon its ubiquitous expression and the view that ENT1 could also be a major player in modulating intracellular nucleoside bioavailability, researchers in the field have considered ENT1 as a sort of housekeeping gene whose deletion can translate into a lethal phenotype. However, the ENT1 knock out mouse is viable, although it shows some phenotypic/biochemical features which are not particularly severe [42].

ENT1 has recently been related to the long ago-described Augustine-negative At^(a–)^ blood type [43]. At^a^ was shown to be a high-frequency antigen. The characterization of the immune complex generated by alloantibodies against this antigen revealed ENT1 as the targeted protein. Indeed, ENT1 appears to be critical for nucleoside homeostasis and erythropoiesis and is abundant in erythrocytes [44,45]. Homozygosity for the p.E391K ENT1 variant is responsible for this rare blood type in people of African ancestry. This non-conservative substitution in the fifth extracellular loop of the protein probably results in the structural disruption of the antigen epitope. However, this substitution does not alter ENT1 biological function. Therefore, these individuals would not present any particular phenotype beyond this unusual blood type. This was not the case in three siblings of European ancestry, homozygous for a null mutation (c.589+1G>), who completely lacked the ENT1 protein [43,45]. These siblings were identified as At_null_. Although no severe phenotype was observed, aging was associated with a progressive ectopic calcification of the bone joints [43]. Interestingly, this feature has also been reported in Ent1 knock out mice [46,47]. The mechanisms behind these alterations are not well-known, but point to adenosine signaling. Indeed, purinergic signaling via P1 receptors, particularly A1 and A2b, has been shown to promote osteogenic differentiation of mesenchymal stem cells [48]. Therefore, in ENT1 null mice and humans, tonic agonistic activation of these receptors might be expected as a consequence of increased extracellular adenosine concentrations. ENT1 and ENT2 can translocate adenosine with similar affinities, but the steady-state plasma concentrations of this nucleoside are strikingly different between both knock out mouse models [49]. Whereas wild type mice show plasma adenosine concentrations of around 1 µM, mean levels in Ent1 and Ent2 knock out mice are 11 µM and 2 µM, respectively. This would favor the hypothesis that ENT1 plays a more relevant role than ENT2 on adenosine homeostasis [25]. It is still an open question as to what the compensation mechanisms that prevent a severe phenotype of ENT1 null individuals are. As already mentioned, ENT1 appears to be crucial for erythropoiesis and, at least in two out of the three ENT1 null siblings described above, a compensatory mechanism would rely upon loss-of-function mutations found in the ABCC4 gene [45]. The rationale for this is that ENT1 appears to be critical for cAMP homeostasis and for the regulation of erythroid transcription factors such as GATA1 and GATA2 [44]. It has been suggested that ENT1 function somehow modulates intracellular cAMP levels. ABCC4 encodes the efflux pump MRP4, whose major endogenous substrate is cAMP [50] and loss-of-function mutants would preserve intracellular cAMP pools.

On the other hand, ENT1 and ENT2 can form homo- and hetero-oligomers, which can translocate to and from the plasma membrane in a regulated manner [51]. Therefore, we cannot rule out the possibility that in terms of overall nucleoside supply for salvage purposes, ENT2 could significantly compensate for ENT1 loss by increasing the half-life residence time of ENT2 homo oligomers at the plasma membrane.

Regarding the compensation of the lack of ENT1 at the inner mitochondrial membrane, one possibility relates to the fact that ENT3 has been suggested to be expressed in the mitochondria [32]. Nevertheless, although ENT3 selectivity significantly overlaps with that of ENT1, the apparent Km values for natural nucleosides are in the millimolar range [30]. Moreover, its function is pH-dependent, with a peak activity at pH 5.5 and a nearly total lack of function at 7.4 [30]. There is an alternative explanation for metabolic compensation in ENT1 null cells. This provides evidence that mitochondria express a deoxynucleotide transporter which would bypass ENT1 function [8,9]. Indeed, oral deoxynucleoside supplementation reverses mitochondrial DNA depletion in MDDS patients harboring mutations in TK2 and Tk2^–/–^ mice as well. As mentioned above, TK2 is a mitochondrial matrix nucleoside salvage enzyme, and improvement following deoxynucleoside therapy is consistent with the idea that pyrimidine deoxynucleotides synthesized by cytosolic kinases (i.e., TK1) can be transported into the mitochondria, thereby bypassing TK2 but also ENT1 [8,9].

**Figure 3:**
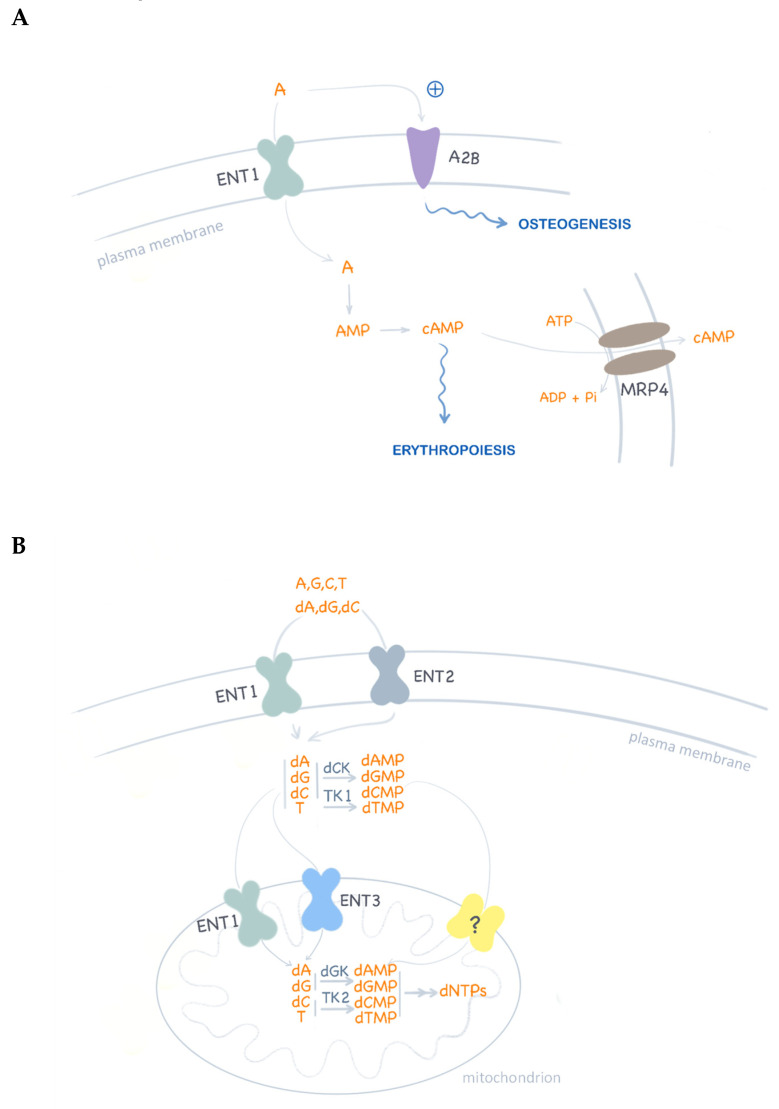
ENT1 functions in nucleotide metabolism. (**A**) Schematic representation of adenosine (A) translocation and signaling. Adenosine activation of P1 receptors (i.e., A2B) promotes osteogenic differentiation of mesenchymal stem cells. Intracellular cAMP levels are modulated by ENT1-associated adenosine translocation and also by MRP4 cAMP efflux. In turn, cAMP homeostasis is critical for erythropoiesis regulation. (**B**) Nucleoside uptake at the plasma membrane is mediated by ENT1 and ENT2 homo and hetero-oligomeric complexes. ENT1 has been reported to translocate nucleosides and deoxynucleosides in mitochondria. In ENT1 null cells, deoxynucleoside supply to mitochondria may be maintained by ENT3 function, although the proven functional expression of an unknown deoxynucleotide transporter (?) may be the best compensatory mechanism to maintain mitochondrial deoxynucleotide pools for DNA replication.

Various regulatory/metabolic links implicating ENT1 likely explaining pathogenic events but also probable compensatory mechanisms for ENT1 loss are schematically shown in Figure 3.

ENT1 deficiency has not been listed in the OMIM catalog yet. In any case, considering the clinical phenotype of the first three human ENT1 null cases and the characteristics of the ENT1 knock out mouse model, it is evident that screening for mutations within the *SLC29A1* gene is warranted in patients with idiopathic disorders characterized by ectopic mineralization.

### 3.2. Genetic Alterations of ENT3. Histiocytosis-Lymphoadenopathy Plus Syndrome. OMIM#602782

Human ENT3 was cloned by Baldwin and colleagues in 2005 [30]. ENT3 possesses a N-terminal hydrophilic domain that bears endosomal/lysosomal targeting motifs. Indeed, when this domain was deleted, truncated ENT3 was mistargeted to the plasma membrane, thereby allowing the proper characterization of ENT3 substrate selectivity and specificity. ENT3 activity was shown to be strongly dependent on pH, with a peak activity at 5.5, suggesting it could be expressed in acidic intracellular compartments, such as lysosomes. Indeed, when permeabilized HeLa cells were checked for endogenous ENT3 expression, an intracellular punctate pattern was observed with some co-localization with CD63, a late endosomal/lysosomal marker [30]. ENT3 has also been reported to be expressed in mitochondria and its gene silencing using specific siRNAs resulted in a significant decrease in the mitochondrial transport of adenosine and guanosine and several nucleoside-derived drugs known to induce mitochondrial toxicity [32]. Although ENT3 in lysosomes would facilitate nucleoside efflux to the cytosol, it is suggested that the relatively acidic pH in the mitochondrial intermembrane space would facilitate the opposite substrate flux, from cytosol to the matrix [31].

Pathogenic variants in the ENT3 encoding gene *SLC29A3* result in different clinical manifestations which correspond to several recessive autosomic disorders previously considered to be distinct, but sharing histiocytosis as a common feature (Table 1). They are all classified under the general name histiocytosis-lymphoadenopathy plus syndrome (OMIM#602782). H syndrome, a genodermatosis with various system manifestations, was the first disorder reported to be caused by ENT3 missense variants [52]. The name “H” syndrome stands for the “H” in its clinical features: hyperpigmentation and hypertrichosis, hepatosplenomegaly, heart anomalies, hearing loss, hypogonadism, low height and hyperglycemia. Other syndromes caused by *SLC29A3* mutations include pigmented hypertrichotic dermatosis with insulin-dependent diabetes (PHID) [53], Faisalabad histiocytosis (FH) [54], and Familial Rosai–Dorfman Disease [54]. Interestingly, a form of osteopetrosis known as dysosteosclerosis has also been reported to be caused by ENT3 variants, suggesting a role for this transporter in osteoclast differentiation [55]. A first set of seven variants (five missense and three deletions resulting in C-terminal truncation) associated with H and PHID syndromes were checked for transporter life cycle and function and revealed dramatic reductions in transport activity (totally absent in the truncated forms) and protein half-life [56]. The number of identified patients and pathogenic variants rapidly increased after the first molecular diagnosis of H and other syndromes [57]. At the molecular level, the recent crystallization of the human ENT1 protein has allowed the mapping of key *SLC29A3* missense variants on their corresponding position in ENT1 structure as a way to understand the mechanistic basis for dysfunction [40,58]. For instance, the p.G437R variant seems to be trafficked normally to the plasma membrane [56], but the mutation maps to the TM10-11 loop where one of the putative pH-sensitive residues is located. p.G437R ENT3 shows decreased Vmax and increased Km values compared to the wild type [56]. Interestingly, this variant has been found in H, PHID and FH patients [59]. Nevertheless, not all genetic variants described so far are missense. Indeed, the first H syndrome patient we identified in Spain [60] showed a novel variant at that time, c.[243delA], which had been concomitantly described by others while studying two siblings with granulomatous histiocytosis [61]. A frameshift deletion in the DNA sequence resulted in the translation of an otherwise non-coding mRNA splice variant whose product was a stable ENT3 protein with decreased but still significant transport activity [61]. Although the two siblings showed a rather mild form of the disorder that could be explained by residual ENT3 function, the third patient bearing the same mutation showed a much more severe phenotype and died at the age of 20. Therefore, it seems quite evident that molecular alterations by themselves might not be the key clue to understand the heterogeneity of clinical features in this group of syndromes.

The involvement of mitochondria in the primary etiology of these syndromes is unclear. The depletion of ENT3 expression in β-cell-derived cell lines impairs mitochondrial function and promotes apoptosis, which could explain the insulin-dependent diabetes reported in PHID and other syndromes [65]. Immortalized B-lymphoblastoid cell lines and skin fibroblasts from the c.243delA H syndrome patient described above have been checked for mitochondrial dysfunctions, but results supporting a mitochondrial involvement are not evident [60]. The enzyme activity of the respiratory chain complexes and mtDNA copy number were not altered in these samples compared to controls. When patient fibroblasts were challenged with low- and high-toxicity doses of several nucleoside analogs known to induce mitochondrial toxicity, no differences in mtDNA copy number were found either [60], although a slight protection against gemcitabine-induced cytotoxicity was found in the patient fibroblasts lacking ENT3 function compared to wild type controls [60]. Interestingly, ENT3 has been identified as a mediator of remdesivir-triggered mitochondrial toxicity [66]. Remdesivir is an antiviral nucleoside-derived prodrug shown to induce the inhibition of mitochondrial respiratory activity and cytotoxicity. This effect was significantly mitigated after the down-regulation of ENT3 expression [66].

As mentioned above, both ENT1 and ENT3 have been reported to be expressed in mitochondria. Therefore, ENT1 could compensate for ENT3 loss of function in ENT3 null patients (Figure 3), at least when dealing with the supply of natural nucleosides. Indeed, some nucleoside analogs which induce mitochondrial toxicity are good ENT1 substrates., However, blocking specifically ENT1 and not ENT3 function with NBMPR induces a moderate reduction in intracellular remdesivir accumulation, which suggests that ENT3 but not ENT1 mediates remdesivir-induced cytotoxicity [67]. Thus, compensation between ENT subtypes may not apply to all cytotoxic drugs, but likely applies to natural substrates. Accordingly, mitochondria may not play a major role in the primary etiology of these syndromes due to cross-compensation between ENT1 and ENT3 mitochondrial transporters.

Many lysosomal solute carrier transporters have been associated with a significant number of diseases and, among them, with this group of syndromes caused by ENT3 variants [68]. Indeed, the study of the phenotype of Ent3^–/–^ mice provided the first robust evidence that ENT3 deficiency could cause a lysosomal storage disease (LSD) [69,70]. Macrophages from Ent3^–/–^ mice showed lysosomal nucleoside accumulation and increased pH, thereby altering lysosomal integrity. These mice recapitulated the histiocytosis clinical phenotype, and this was the consequence of altered macrophage function and accumulation. Using this animal model, it was later shown that ENT3 is relevant to the maintenance of peripheral T cell homeostasis and regulates lysosome integrity and abundance and nucleoside availability as well [71]. Interestingly, autophagy is impaired in ENT3^–/–^ T cells, resulting in the accumulation of vacuoles but also of disorganized mitochondria which cannot be properly cleared off. It is likely that the altered mitochondrial function reported in β cells and suggested to be responsible for the diabetic phenotype in some of these patients [65] might also be the result of altered autophagy processes. Therefore, it seems that there is a mitochondrial involvement in the etiology of the disease, but this may not be a primary causative event but rather secondary to lysosomal dysfunction.

**Figure 4:**
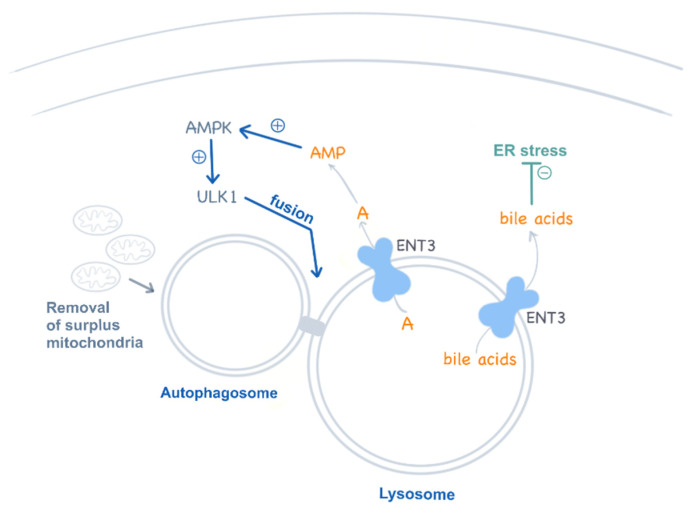
ENT3 functions in lysosomes. ENT3 is proposed to regulate autophagy by modulating adenosine (A) release from the lysosomes into the cytosol, which contributes to AMPK activation and the regulation of the AMPK–mTOR–ULK axis. Additionally, ENT3 has been recently proposed as a low-affinity bile acid transporter at pH 5.5, contributing to ER stress regulation.

The recent observation that ENT3 deficiency impairs multipotent stem cells is a major breakthrough in the understanding of the etiology of this disease [70]. Mesenchymal and hematopoietic stem cells also show autophagy disturbances which compromise their differentiation into their corresponding lineages, a feature that contributes to explaining the broad systemic impact of this deficiency in these mice but also in humans. Interestingly, this study also provided evidence that ENT3 in lysosomes releases adenosine into the cytosol, which in turn activates AMPK and regulates autophagy via the AMPK–mTOR–ULK axis [70] (Figure 4). This pathway is impaired in ENT3 null cells. As for T cells, there is also a mitochondrial involvement in stem cell disturbances. *Slc29a3*^–/–^ mesenchymal stem cells show impaired mitochondrial respiration and metabolic performance (i.e., β-oxidation of fatty acids) [70].

Amazingly, one of the most unexpected turns in the field of nucleoside transporters comes from the recent results, published by the same group, showing that ENT3 may be a low-affinity bile acid transporter at an acidic pH (5.5) but not at 7.4 [72] (Figure 4). Thus, ENT3 would favor bile acid efflux from lysosomes, although bile acids would be sequestered within these organelles if a functional ENT3 was lacking. Bile acids have been shown to protect hematopoietic stem cells from ER stress [73]. Accordingly, these authors suggest that this function would be impaired in ENT3-deficient cells, a feature that would impact, in particular, erythropoiesis [72].

The understanding of the etiology of this syndrome paves the way for the discovery of suitable therapies for patients. Stem cell transplantation and AICAR treatment reduced ENT3 knock out mouse disease alterations [70]. AICAR is a potent AMPK activator, well tolerated by humans [74], that would overcome the reduced adenosine-dependent AMPK activation reported in stem cells from ENT3 null mice. On the other hand, a significant number of patients develop autoinflammatory complications, and inflammatory-related pathways are dysregulated in immune system cells from H and Rosay–Dorfman syndrome patients [75]. Although only a few case reports are available in the literature, it seems likely that targeting the inflammatory component of the disease would result in some clinical improvement [76,77,78].

### 3.3. Genetic Alterations of CNT1. Uridine-Cytidineuria (URCTU). OMIM#618477

**Figure 5:**
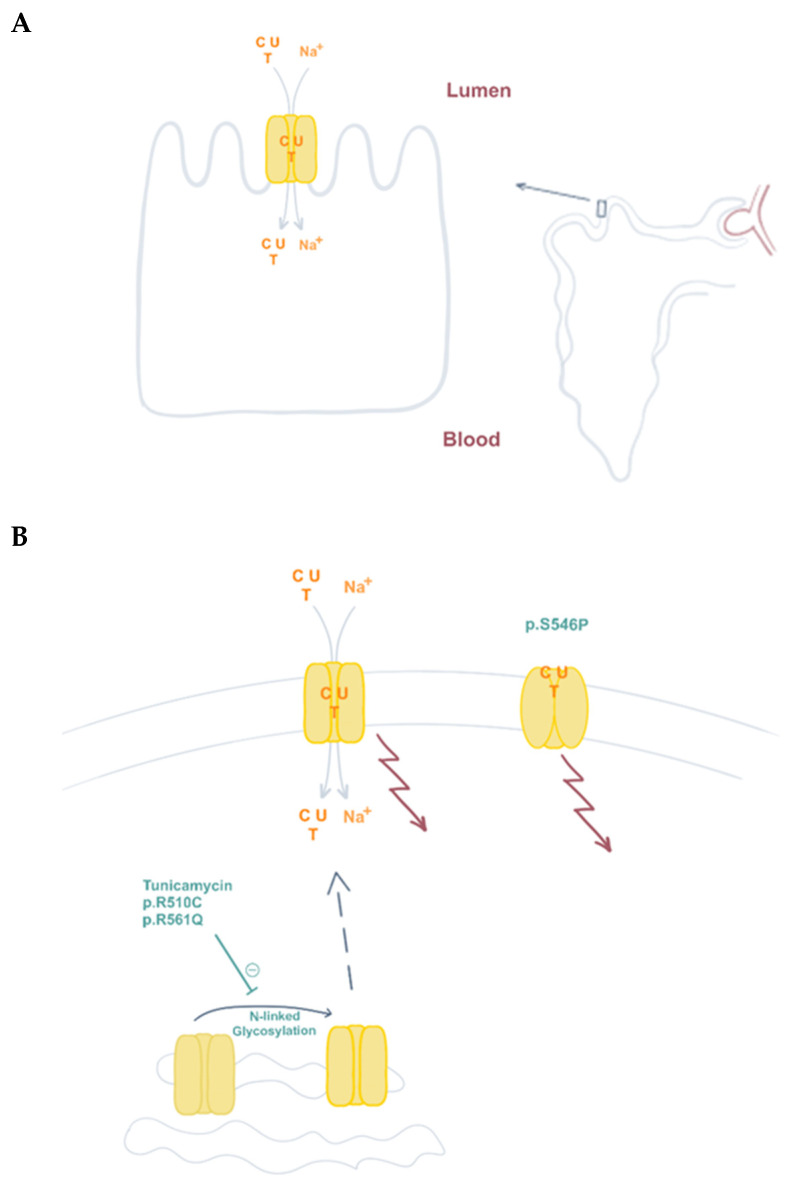
CNT1 function and presence at the plasma membrane contribute to pyrimidine reabsorption in the kidney. (**A**) CNT1 is expressed at the apical plasma membrane of kidney proximal tubule epithelial cells, where it is responsible for pyrimidine reabsorption (C, cytidine; U, uridine; T; thymidine). (**B**) N-linked glycosylation contributes to CNT1 plasma membrane localization. This post-translational modification of the protein is partially impaired in variants p.R510C and p.R561Q, causative of URCTU. CNT1 also functions as a transceptor. Besides pyrimidine translocation, it can also induce changes in signaling pathways (arrows, see text), regardless of its transporter function. Variant p.S546P lacks this translocation ability, but is properly located at the plasma membrane and retains some wild type transceptor functions.

URCTU is the only clinical condition reported so far caused by pathogenic variants in a member of the *SLC28* gene family, the *SLC28A1* gene, encoding the high-affinity pyrimidine concentrative nucleoside transporter CNT1. Our laboratory identified the first case of URCTU in a newborn child showing very high pyrimidineuria levels [63]. The uridine concentration in urine from this patient, at the age of 4 weeks, was 100 mmol/mol creatinine, whereas the age-matched reference range for controls is 0.06–2.5 mmol/mol creatinine. Urine cytidine levels were also increased, although to a lesser extent than uridine. Plasma pyrimidine nucleoside concentrations were not particularly altered in this child. This patient had been previously screened at the molecular level for all known gene alterations impacting on pyrimidine metabolism, but no pathogenic mutations were identified. The pyrimidineuria pointed towards a defect in pyrimidine nucleoside reabsorption as a possible cause for this biochemical defect. As mentioned above, polarized insertion of CNTs in (re)absorptive epithelia is crucial to allow the vectorial flux of nucleosides across them. CNTs are located at the apical membrane in these epithelia. Moreover, CNT1 is highly expressed in the proximal convoluted tubule (PCT) of the nephron, a segment bearing most of the solute transporters allowing the reabsorption, among others, of glucose, amino acids, and nucleosides [38] (Figure 5). This patient expressed two missense variants in hCNT1, p.R510C and p.R561Q. The former had previously been described, and although it shows low allelic frequency in Europeans, it is relatively frequent in the Asian population. Based upon the CNT structural modeling we previously developed [79], the p.R510 is located at the extracellular side of the protein and the p.R561 faces the cytosol. Neither are close to the binding and translocation domains, but both variants might generate changes in the residue interaction pattern and the residue-occupied volume, possibly impacting on tertiary structure stabilization [63]. Interestingly, the patient was a compound heterozygote for each individual variant, and alleles were in a trans disposition. For informative purposes, the double mutant was generated and checked for activity and intracellular processing. p.R510C and R561Q showed a dramatic decrease in transport activity and altered post-translational processing, basically due to impaired glycosylation. Our analysis also demonstrated that glycosylation was crucial for proper CNT1 insertion and maintenance at the plasma membrane and for biological function as well (Figure 5B). CNTs have been reported to be homotrimers. Therefore, we checked the functional impact of co-expressing both hCNT1 proteins, p.R510C and p.R561Q. Co-expression also resulted in decreased activity, although the impairment of CNT1 function was not as dramatic as that found in the double mutant [63]. This patient also suffered from perforin deficiency, a severe condition which could by itself contribute to the multi-organ failure observed in this child, conforming with a lethal phenotype [80]. Therefore, it is difficult to figure out what the phenotype of CNT1 deficiency alone would be. Two more URCTU patients were reported in the literature a few months later [64]. The first one was a child with significant pyrimidineuria who also showed a pathogenic variant in PPRT2, a gene that helped to explain the epileptic phenotype he developed in his infancy. He and his older brother displayed a homozygous missense variant in the CNT1 encoding gene which resulted in a non-conservative amino acid residue change (p.S546P). Beyond the biochemical phenotype (pyrimidineuria), both were described as clinically asymptomatic. Indeed, when CNT1 deficiency was later indexed in the OMIM catalog as URCTU (OMIM#618477), this inborn error of metabolism was considered to be, putatively, a benign metabolic phenotype. Nevertheless, we believe that this is arguable.

Serine substitution by proline at position 546 was initially reported in the screening of a panel of 24 plasma membrane transporter genes performed by a NIH-funded consortium back in 2003 [81]. More than 200 DNA samples from ethnically diverse non-related individuals were screened for genetic heterogeneity in selected drug transporters likely to be relevant to pharmacogenetics. This variant showed very low allelic frequency and was only identified in African Americans [82]. A preliminary functional characterization showed that this substitution resulted in CNT1 loss of function [82]. This seems to agree with the idea that CNT1 deficiency is a benign condition, considering that the DNA bank was presumably built up with samples from healthy individuals. We further characterized this variant, and although we confirmed its loss of function, we observed that the mutated protein could properly be expressed, folded, and inserted at the plasma membrane [83]. Moreover, p.S546P, when expressed in epithelial MDCK cells grown on transwells, was effectively trafficked to the apical membrane without any apparent missorting. This makes a difference with the first patient we identified, because in that case, CNT1 function was partially preserved but the protein showed impaired processing and life-cycle disturbances [63].

CNT1 protein expression is lost in many of the epithelial tumors analyzed so far [84,85,86]. Restitution of CNT1 protein expression in pancreatic adenocarcinoma cell lines resulted in cell cycle arrest, non-apoptotic cell death, the inhibition of cell migration, and PARP hyperactivation, among other effects [87]. Interestingly, when we restored CNT1 protein expression but used a non-functional transporter protein (we used indeed the p.S546P mutant for that purpose), the cellular effects we observed were identical to those found after restoring the wild type CNT1 protein. This opens up the possibility that CNT1 may have a transceptor role, which means that CNT1 (and maybe other NT proteins) may have a biological role beyond the mere supply of nucleosides which could be eventually compensated by other NT subtypes [88] (Figure 5B). This basic knowledge regarding CNT1 biology implies that the two siblings mentioned above bearing this mutation are asymptomatic because key functions unrelated to nucleoside transport but requiring transporter structural integrity are preserved. Therefore, we think that it is still possible that URCTU could develop with additional clinical features beyond a mere biochemical urine disturbance. It is not clear to us as to what the expected phenotype of severe cases of CNT1 deficiency could be, but the measurement of pyrimidine levels in urine may be relatively feasible in a routine manner and could be eventually implemented when facing undiagnosed clinical cases.

## 4. Concluding Remarks

Diseases caused by alterations in genes encoding the protein machinery implicated in nucleotide metabolism, although rare, have been shown to impact in all pathways responsible for the maintenance of nucleotide homeostasis, including membrane transporters. At this moment, three genes encoding nucleoside and nucleobase transporter proteins have been reported to be mutated in humans, *SLC29A1*, *SLC29A3*, and *SLC28A1*, impairing the expression and function of ENT1, ENT3, and CNT1, respectively. Phenotype heterogeneity among patients is high, particularly in ENT3 deficiency, the best characterized dysfunction so far. However, heterogeneity cannot be merely explained at the molecular level, although recent progress in the knowledge regarding NT structures helps to explain the mechanistic basis for disturbances in the transport activity of mutants. Indeed, progress in the understanding of ENT1 and CNT3 structures are major breakthroughs in the field [40,89]. However, we think that clinical heterogeneity might also depend on the complex physiological networks to which these transporter proteins functionally contribute. Indeed, some features of the etiology of these diseases can be explained, at least in part, by the current knowledge on NT biology, although more basic knowledge on NT functional and regulatory properties is still needed.

It is to some extent surprising that, in general trends, genetic alterations impacting on these transporter proteins do not appear to result in clinical phenotypes similar to those associated with enzyme deficiencies implicated in nucleotide metabolic pathways. Although novel rare diseases and clinical phenotypes associated with nucleoside and nucleobase transporter disturbances might still be unveiled in the near future, current knowledge suggests that those identified so far do not have a deleterious impact on salvage processes and are metabolically compensated somehow. Nevertheless, there is still the possibility that some of them develop with biochemical alterations, as shown for URCTU, which suggests that metabolic screenings focusing on purines and pyrimidines in biological fluids might be useful as a first approach to diagnose suspected nucleoside transporter-related rare diseases.

## Figures and Tables

**Table 1:** Nucleoside transporter genetic variants and associated diseases.

Syndrome	Gene (Location)	Protein	Variant	Heredity	Clinical Features	Reference
Augustine-null blood type	*SLC29A1* (6p21.1)	hENT1	c.1171G>A (p.E391K)	-	Rare blood type (At^a^)	[43]
			c.589+1G>C	AR	At_null_—progressive ectopic calcification of bone joints	[43,45]
Histiocytosis- lymphadenopathy plus syndrome (OMIM #602782) entry 3	*SLC29A3* (10q22.1)	hENT3	p.G427S p.G437R c.1045delC	AR	H syndrome: hyperpigmentation and hypertrichosis, hepatosplenomegaly, heart anomalies, hearing loss, hypogonadism, low height and hyperglycemia	[57]
			p.M116R c.940delT p.G437R p.E444X p.T449R	AR	Pigmented hypertrichotic dermatosis insulin-dependent diabetes (PHID)	[53]
			c.300+1G>A	AR	Faisalabad histiocytosis (FH)	[54]
			p.G437R	AR	Familial Rosai-Dorfman disease (RDD)	[54]
			p.G437R p.F103X	AR	Familial sinus histiocytosis with massive lymphadenopathy (SHML)	[62]
			p.S203P;R386Q p.T449R	AR	Dysosteosclerosis (osteoporosis)	[54]
Uridine-cytidineuria, URCTU (OMIM #618477)	*SLC28A1* (15q25.3)	hCNT1	p.R510C;R561Q	CH	Uridine-cytidineuria	[63]
			p.S564P	AR	Elevated urinary excretion of uridine and cytidine	[64]

AR, autosomal recessive; CH, compound heterozygosity.
